# Tributyrin Attenuates Metabolic and Inflammatory Changes Associated with Obesity through a GPR109A-Dependent Mechanism

**DOI:** 10.3390/cells9092007

**Published:** 2020-09-01

**Authors:** Fabio Takeo Sato, Yu Anne Yap, Amanda Rabello Crisma, Mariana Portovedo, Gilson Masahiro Murata, Sandro Massao Hirabara, Willian Rodrigues Ribeiro, Caroline Marcantonio Ferreira, Maysa Mariana Cruz, Joice Naiara Bertaglia Pereira, Tanyara Baliani Payolla, Suzana Eiko Sato Guima, Andrew Maltez Thomas, João Carlos Setubal, Maria Isabel Cardoso Alonso-Vale, Marinilce Fagundes Santos, Rui Curi, Eliana Marino, Marco A. R. Vinolo

**Affiliations:** 1 Department of Genetics, Evolution, Microbiology and Immunology, Institute of Biology, University of Campinas, Campinas 13083007, Brazil; fabiotakeosato@gmail.com (F.T.S.); mariportovedo@gmail.com (M.P.); 2 Department of Biochemistry, Biomedicine Discovery Institute, Monash University, Melbourne 3800, Australia; yu-anne.yap@monash.edu; 3 Department of Clinical Analyses, Federal University of Paraná, Curitiba 80060000, Brazil; amycrisma@yahoo.com.br; 4 Department of Physiology and Biophysics, Institute of Biomedical Sciences, University of São Paulo, São Paulo 05508000, Brazil; gilmasa@gmail.com (G.M.M.); tany_line@yahoo.com.br (T.B.P.); ruicuri59@gmail.com (R.C.); 5 Interdisciplinary Postgraduate Program in Health Science, Cruzeiro do Sul University, São Paulo 01506000, Brazil; sandromh@yahoo.com.br (S.M.H.); joice_naiarabp@hotmail.com (J.N.B.P.); 6 Department of Pharmaceutics Sciences, Institute of Environmental Chemistry and Pharmaceutical Sciences, Federal University of São Paulo, Diadema 09972270, Brazil; will.ribeiro181@hotmail.com (W.R.R.); caro_valpa@hotmail.com (C.M.F.); 7 Department of Biological Sciences, Institute of Environmental, Chemical and Pharmaceutical Sciences, Federal University of São Paulo, Diadema 09972270, Brazil; maysamariana@gmail.com (M.M.C.); alonsovale@gmail.com (M.I.C.A.-V.); 8 Department of Biochemistry, Institute of Chemistry, University of São Paulo, São Paulo 05508000, Brazil; suzy.eiko@gmail.com (S.E.S.G.); andrewmaltezthomas@gmail.com (A.M.T.); setubal@iq.usp.br (J.C.S.); 9 Department of Cell and Developmental Biology, Institute of Biomedical Sciences, University of São Paulo, São Paulo 05508000, Brazil; mfsantos@usp.br; 10 Butantan Institute, São Paulo 05503900, Brazil; 11 Experimental Medicine Research Cluster (EMRC), University of Campinas (UNICAMP), Campinas 13083007, Brazil; 12 Obesity and Comorbidities Research Center (OCRC), University of Campinas (UNICAMP), Campinas 13083864, Brazil

**Keywords:** butyrate, microbiota, insulin resistance, dysbiosis

## Abstract

Obesity is linked with altered microbial short-chain fatty acids (SCFAs), which are a signature of gut dysbiosis and inflammation. In the present study, we investigated whether tributyrin, a prodrug of the SCFA butyrate, could improve metabolic and inflammatory profiles in diet-induced obese mice. Mice fed a high-fat diet for eight weeks were treated with tributyrin or placebo for another six weeks. We show that obese mice treated with tributyrin had lower body weight gain and an improved insulin responsiveness and glucose metabolism, partly via reduced hepatic triglycerides content. Additionally, tributyrin induced an anti-inflammatory state in the adipose tissue by reduction of *Il-1β* and *Tnf-a* and increased *Il-10*, Tregs cells and M2-macrophages. Moreover, improvement in glucose metabolism and reduction of fat inflammatory states associated with tributyrin treatment were dependent on GPR109A activation. Our results indicate that exogenous targeting of SCFA butyrate attenuates metabolic and inflammatory dysfunction, highlighting a potentially novel approach to tackle obesity.

## 1. Introduction

Obesity is a chronic condition characterized by an abnormal or excessive accumulation of fat associated with a low-grade systemic inflammatory tone and risk to health. According to a World Health Organization report in 2016 [1], 13% of the world adult population was obese (a body mass index (BMI) of more than 30 kg/m^2^). In some countries such as the United States, 70% of the population is overweight (BMI ≥ 25 kg/m^2^) and 38% is obese [2]. Overweight and obesity are also risk factors for other diseases such as type 2 diabetes mellitus (T2DM), hypertension and cardiovascular diseases, which are leading causes of death [3]. 

Increased local or systemic levels of proinflammatory mediators are observed in obese individuals, such as tumor necrosis factor (TNF)-α and interleukin (IL)-1β [4,5]. These contribute to the impairment of multiple organs including the adipose tissue, pancreas, liver and skeletal muscle, which may underlie the link between obesity and the associated diseases [4,5]. Therefore, strategies for the attenuation or reversal of the chronic sustained proinflammatory state present in obese individuals may be a relevant therapeutic approach for restoring homeostasis and the prevention of many systemic diseases. 

Growing evidence supports a central role of the intestinal microbiota in the development of obesity. For example, changes in the composition of the intestinal microbiota are known to occur in obese murine models [6] and humans [7]. Moreover, the transference of a dysbiotic microbiota from obese individuals induces an obese metabolic phenotype in the host [8,9]. Despite these findings, the mechanisms involved in the association between changes in microbiota and obesity are not well understood [10]. 

The short-chain fatty acids (SCFAs) acetate, propionate and butyrate are products of diet metabolization by gut microbiota. These molecules have critical metabolic effects on the host, and changes in their production have been associated with obesity development [11]. SCFAs are energetic substrates for intestinal cells, hepatocytes and other tissues. They also regulate metabolism in multiple organs through, for example, their effects on hormone production, as the case of glucagon-like peptide-1 (GLP-1), and via their actions on the central nervous system and brown adipose tissue [11]. Additionally, these bacterial metabolites modulate the inflammatory response and activation of immune cells [12]. Indeed, several studies have demonstrated a remarkable and beneficial role for SCFA-based dietary interventions in the pathogenesis of inflammatory diseases, such as allergic conditions, asthma, arthritis, colitis, kidney disease, hypertension, type 1 diabetes mellitus (T1DM) and intestinal infection [13,14,15,16,17,18,19,20]. 

In line with previous findings, SCFAs have a beneficial influence on hepatic metabolism by preventing the progression of nonalcoholic fatty liver disease (NAFLD), T2DM and insulin resistance (IR) in rodents [21,22] and humans [23,24]. Similarly, studies have demonstrated that SCFAs are key factors in reducing and preventing body weight gain and obesity [25,26]. In skeletal muscle, SCFAs function in two ways. First, there is a low supply of lipids due to the positive effect of SCFAs on adipose tissue lipid storing capacity and a consequent reduction of inflammatory cytokines and the prevention of IR [27]. Secondly, SCFAs directly increases fatty acid oxidation in muscle by stimulating the AMP-activated protein kinase (AMPK) signaling [27] and inducing the expression of metabolic genes like peroxisome proliferator-activated receptor (PPAR) gamma coactivator 1-alpha (PCG1α) and peroxisome proliferator-activated receptor delta (PPAR-δ) [28,29]. 

We and others have shown that individuals suffering T1DM and T2DM have a deficiency in fecal and circulating microbial SCFAs [13,30,31,32]. Elevation of butyrate availability through increased dietary fiber intake or oral supplementation prevents the development of metabolic and inflammatory changes associated with high-fat diet (HFD)-induced obesity via improvement in glucose homeostasis, and insulin sensitivity in mice [28,33,34,35,36,37,38]. Similarly, we have shown that the administration of a prodrug of butyrate, tributyrin (Tb), prevents the development of metabolic and inflammatory alterations in HFD-fed mice [39]. Tb consists of three butyrate molecules esterified to glycerol that is hydrolyzed by lipases. Tb presents better pharmacokinetic and lower toxicity than butyrate [40]. Additionally, Tb was found to attenuate some of the deleterious changes associated with HFD intake in mice, including adipose tissue inflammation, hepatic steatosis and IR [39]. Considering these positive effects, we have tested the efficacy of Tb treatment in mice that already present obesity associated-metabolic and inflammatory alterations. In addition, we investigated the role of the G-protein coupled receptor 109A (GPR109A) in the mechanism of action of this lipid.

## 2. Materials and Methods

### 2.1. Animals

Male (6 to 8 weeks old) adult C57BL/6 mice were purchased from the Multidisciplinary Center for Biological Research (CEMIB) or obtained from the Animal Research Platform (Monash University). Animals were maintained in an animal facility using a light/dark cycle of 12 h/12 h, at constant temperature (22 °C), with water and food ad libitum and constant temperature (23 °C). Experiments with GPR43 (*Gpr43*^−/−^) and GPR109A (*Gpr109a*^−/−^) knockout mice were performed at Monash University (Melbourne, AU). These mice strains and their controls (WT) were obtained from the Monash Animal Research Platform (Melbourne AU). *Gpr43*^−/−^ and *Gpr109a*^−/−^ mice were previously described [16,41]. Experimental protocols were approved by the Ethics Committee for Animal Use (CEUA, Unicamp, protocol number: 2934-1) and by the Animal Care Committee of the School of Biological Sciences at Monash University (MARP/2015/126).

### 2.2. Experimental Design and Diets

The high-fat diet (HFD) used in this study provides 26% of its energy from carbohydrates, 59.1% from fat (lard) and 14.9% from protein. A control diet (CD) that provided 75.8% of its energy from carbohydrates, 9.5% from fat and 14.7% from protein was also used in the study. Both diets were previously described [39,42] and contained the same amount of fiber (50 g/kg of cellulose). Mice were maintained on the HFD for eight weeks. After that, they were weighed and randomly divided into two experimental groups. One group received 2.0 g/kg Tb by gavage three times/week at intervals of 48 h for six weeks, while the other received water by gavage for the same period. Body weight and food intake were monitored during the whole protocol. At the end of the 14-week procedure, mice were fasted 6 h and humanely euthanized using isoflurane (Cristália, Itapira, Brazil), followed by cervical dislocation. Biological samples were harvested and immediately processed or stored at −80 °C. The same procedures were also performed with C57BL/6 (wild-type (WT)), *Gpr43*^−/−^ and *Gpr109a*^−/−^ mice at Monash University (Melbourne, AU) using commercial HFDs with 43% or 58% of energy from fat (SF04-001 and SF17-232, Specialty Feeds, Glen Forrest, Australia). These two diets contained 58 and 50 g/kg of fiber (cellulose), respectively.

### 2.3. Oxygen Consumption/Carbon Dioxide Production

The metabolic activity of mice was evaluated by indirect calorimetry using CLAMS (OxymaxLab Animal Monitoring System, Columbus, OH, USA) for 24 h after 24 h of acclimatization to the calorimetry cages. The ratio of respiratory exchange was measured using the VCO_2_/VO_2_ ratio. Calorimetry was performed in the 12th week of the protocol.

### 2.4. Serum Analyses

Serum triglycerides, cholesterol, low-density lipoprotein (LDL) and high-density lipoprotein (HDL) concentrations and alanine (ALT) and aspartate (AST) transaminases activities were analyzed using commercial kits (Labtest Diagnóstica SA, Minas Gerais, Brazil and Randox, Count Antrim, UK). Nonesterified fatty acids (NEFA) were measured using the HR Series NEFA-HR (2) with NEFA Standard Solution (Wako Chemicals USA, Richmond, VA, USA) or kit from Randox (Randox; Crumlin, UK).

### 2.5. Short-Chain Fatty Acid (SCFA) Measurements

Chromatographic analyses were performed using a Shimadzu 2010 system with CG solution software, equipped with an AOC-20i automatic liquid sampler (Shimadzu), flame ionization detector (FID, Shimadzu) and fused-silica capillary RTX-WAX (Restek Corporation, Bellefonte, PA, USA) with dimensions of 30 m × 0.25 mm internal diameter coated with a 0.25 µm thick layer of polyethylene glycol. The initial oven temperature was held for 2 min. at 100 °C, increased to 110 °C at a rate of 15 °C/min, held 3 min at this temperature, increased to 140 °C at a rate of 10 °C/min, held 1 min and finally increased to 230 °C at a rate of 70 °C/min. The FID temperature was maintained at 260 °C and the flow rates of H_2_, air and the make-up gas N_2_ were 35, 350 and 25 mL/min, respectively. Sample volumes of 1 µL were injected at 260 °C using a split ratio of approximately 25:1. The runtime for each analysis was 12.95 min. Each sample contained 100 µL of serum, 20 mg of NaCl, 10 mg of citric acid, 20 µL of 1 M HCl and 100 µL of butanol. The tubes were vortexed for 2 min and centrifuged at 13,000× *g* for 15 min. The supernatant was transferred to microtubes, and 1 µL was injected into the gas chromatograph. To quantify the acids, a 0.015–1 mg/mL calibration curve was used. The serum concentration of SCFAs was expressed in μg/mL

### 2.6. Glucose Tolerance Test (GTT)

GTT was performed in 6 h fasted mice in the 12th week of the protocol. In brief, 5 µL of blood from the tail vein was collected before (fasting glucose) and after 15, 30, 60, 90 and 120 min of glucose administration (i.p. at 2.0 g/kg) to the mouse. Blood samples were then mixed with 20 µL of 5% solution of trichloroacetic (TCA). After centrifugation, the supernatant was collected and used for glucose measurements with a commercial kit (Labtest Diagnóstica SA, Minas Gerais, Brazil). 

### 2.7. Insulin Tolerance Test (ITT)

ITT was performed in 6 h fasted mice in the 13th week of the protocol. Blood samples were obtained from the tail vein before (0) and after insulin administration (i.p. 0.75 U/kg, Humulin R from Lilly, Indianapolis, USA or Novolin R from Novo Nordisk, Clayton, NC). Samples were deproteinated with TCA and then used for glucose determination. To minimize the effects of the circadian cycle, GTT and ITT were always performed in the afternoon. The rate of glucose disappearance (K_itt_) was calculated using the −0.693/t_1/2_ formula, where t_1/2_ is calculated from the slope of the least-^square^ analysis of the plasma glucose concentration during the linear decay phase. 

### 2.8. Immunophenotypic Characterization of Stromal Vascular Cells 

Approximately 1 g epididymal white adipose tissue (eWAT) was finely minced with scissors and digested in 4 mL of digestion buffer (0.8 mg/mL collagenase type 1 and 4% BSA) for 45 min, at 37 °C, with vigorous shaking. The digestion solution was neutralized with 8 mL of ice-cold 3% FBS RPMI 1640 solution and filtered through a 70 µm cell strainer. Stromal vascular cells (SVCs) were obtained by centrifugation at 290 rcf for 5 min at 4 °C and resuspended in a 5 mL red blood cell (RBC) lysis buffer for 3 min at room temperature with occasional gentle shaking. Two mL of ice-cold FACS buffer was added to neutralize RBC lysis. Cells were centrifuged at 290 rcf for 5 min at 4 °C, resuspended in 2 mL ice-cold FACS buffer, and prepared for flow cytometry analysis. For extracellular staining, cells were stained with the following antibodies: anti-CD45 (30-F11, BD Bioscience, San Jose, CA, USA), anti-CD11c (HL3, BD Bioscience) anti-CD11b (M1/70, BD Bioscience), anti-CD206 (MR5D3, Biolegend, San Diego, CA, USA), anti-F4/80 (BM8, Invitrogen), anti-CD4 (RM4-5, Biolegend) and anti-CD25 (PC61, BD Bioscience). Briefly, cells were incubated with extracellular antibodies for 30 min at 4 °C in the dark. Cells were then centrifuged at 290 rcf for 5 min at 4 °C, washed once with phosphate-buffered saline (PBS) and resuspended in a 200 µL FACS buffer. For intracellular staining, cells were surface stained, washed, fixed/permeabilized and then stained with anti-Foxp3 (MF23, BD Bioscience) using Foxp3/Transcription Factor Staining Buffer Set (eBioscience) according to manufacturer’s instructions. Data for the following populations, F4/80^high^CD11b^high^CD11c^high^ (M1 macrophages), F4/80^high^CD11b^high^CD206^high^ (M2 macrophages) and CD4^+^Foxp3^+^CD25^+^ (regulatory T cells), were collected with a BD LSR Fortessa and analyzed with FlowJo software (Version 10.6.1).

### 2.9. Liver Analysis

Part of the liver was fixed with a 3.7% buffered formaldehyde solution for at least 8 h at room temperature, dehydrated, processed and embedded in Paraplast (Sigma-Aldrich, St. Louis, MO, USA). To evaluate tissue morphology and the degree of liver steatosis, liver sections (7 µm) from three mice of each group (HFD and HFD+Tb) were stained with hematoxylin and eosin and qualitatively analyzed for fat accumulation and inflammation. To measure liver triacylglycerol (TAG) content, liver samples (100 mg) were homogenized in 4 mL of chloroform and methanol solution (2:1) for 16 h at 4 °C in glass tubes. After this period, 2 mL of a solution 0.6% of NaCl was added and the samples were centrifuged at 425 rcf for 20 min. The organic layer was collected and dried for approximately 2 days. Samples were solubilized in 200 µL of isopropanol and quantified using a commercial kit (Labtest Diagnóstica SA, Minas Gerais, Brazil). 

### 2.10. Quantitative Reverse Transcription Polymerase Chain Reaction (RT-PCR)

Total RNA was extracted using Trizol reagent (Invitrogen, Thermo Fisher Scientific, Waltham, MA, USA). RNA was converted to cDNA using the High-Capacity cDNA kit according to the manufacturer’s instructions (Applied Biosystems). PCR was performed using Rotor Gene (Qiagen, Venlo, Netherlands) and a kit containing SYBR Green as a fluorescent dye (Power SYBR Green PCR Master Mix, Applied Biosystems, Foster City, CA, USA). Quantification of gene expression was performed using a ΔΔCt method with ubiquitin C (UBC) and β2-microglobulin as housekeeping genes for eWAT and liver, respectively. The sequences of the primers used are presented in Table S1. To evaluate the best constitutive gene from each tissue analyzed, the Genorm (Version 3.5), an Excel-based software package was used [43].

### 2.11. Sequencing and Bioinformatics Analysis of Fecal 16S rRNA

DNA was extracted from feces and purified using the PureLink Microbiome DNA Purification kit (Invitrogen, Carlsbad, CA, USA). The quantification of the purified DNA was done using Quant-it Pico-Green dsDNA Reagents and Kits (Invitrogen, CA, USA). The amplification and sequencing of 16S rRNA amplicons was performed in the CATG from the Institute of Chemistry, University of São Paulo. For the 16S metagenomic sequencing library preparation, PCR was performed using the following primers S-D-Bact-0341-b-S-17.

(341F, 5′-TCGTCGGCAGCGTCAGATGTGTATAAGAGACAGCCTACGGGNGGCWGCG) and S-D-Bact-0785-a-A-2160 433 (785R, 5′-GTCTCGTGGGCTCGGAGATGTGTATAAGAGACAGGACTACHVGGGTATCTAATC) for amplification of the V3 and V4 variable regions of the 16S rRNA gene. The adapters suggested on the Illumina workflow for 16S Metagenomic Sequencing Library Preparation were also included in the reactions. The DNA polymerase kit used was KAPA HiFi Hotstart Ready Mix (Kapa Biosystems, Wilmington, MA, USA). Two hundred nM of each primer at 95 °C for 3 min followed by 25 cycles at 95 °C for 30 s, 55 °C for 30 s, 72 °C for 30 s and with a final cycle of 72 °C for 5 min. Verification of amplicon size (expected ~550 bp) was performed using the Bioanalyzer DNA 1000 chip (Agilent Technologies, Santa Clara, CA, USA). Removal of PCR contaminants was performed by AMPure XP beads (Beckman Coulter, Inc., Indianapolis, IN, USA). The dual index was attached using Nextera XT Index Kit and after a second round of PCR cleanup was performed with AMPure XP beads. V3–V4 16S indexed amplicon libraries were validated using the Bioanalyzer High Sensitivity DNA chip thorough verification of the expected size of around 630 bp. Analysis of obtained data was performed in several steps. Raw reads were filtered using Prinseq lite v. 0.20.4 [44] with the removal of sequences that had an average quality score lower than 20 (Q20). To remove the primers and adapters, the software used was cutadapt v. 1.14 [45]. After preprocessing (filtering and adapter removal), the sequences were clustered into Operational Taxonomic Units (OTUs) considering a similarity of 97% shared between reads belonging to the same OTU. The UPARSE method [46] included in the USEARCH software (version 8.1.1812) [47] was used for this clustering step, according to a pipeline based on the clustering-first approach from the Brazilian Microbiome Project (BMP) [48]. We plotted rarefaction curves using QIIME (version 1.9.1) [49]. Taxonomy was assigned for each OTU by the RDP classifier [50]. For these analyses, we used Welch’s *t*-test and Benjamin–Hochberg false discovery rate (FDR) correction from STAMP [51]. These analyses were performed at the Bioinformatics Laboratory at the Institute of Chemistry, University of São Paulo. Data from the bacteria DNA sequencing is publicly available at BioProject NCBI (PRJNA641570).

### 2.12. Measurement of Hormones in Serum Samples

The hormones gastric inhibitory polypeptide (GIP), glucagon-like peptide 1 (GLP-1), leptin, pancreatic polypeptide (PP), peptide YY (PYY) and insulin were measured in serum samples using the mouse gut hormone magnetic bead panel kit from Millipore (MGTMAG-78K, Billerica, MA, USA) according to the instruction manual. The reader used was the Luminex MAGPIX (Madison, WI, USA) and the software used to analyze the data was the Millipore MILLIPLEX Analyst 5.1.

### 2.13. Statistical Analysis

Results are presented as the mean ± standard deviation (SD). Comparisons between experimental groups were performed using Student’s *t*-test or Mann–Whitney test, depending on the sample distribution. For multiple group tests, we performed one-way ANOVA and Tukey post-test analysis for multiple comparisons or two-way ANOVA and Bonferroni’s post-test. *p* < 0.05 was considered statistically significant. Data presented were obtained from at least two independent experiments.

## 3. Results

### 3.1. Tb Reverses the Biochemical and Metabolic Patterns Associated with Obesity

**Figure 1:**
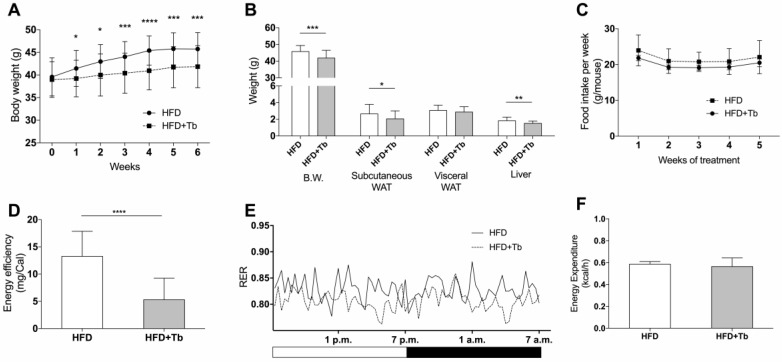
Tributyrin (Tb) treatment reduced body weight gain in obese mice. (**A**) Measurement of body weight of mice during the treatment. (**B**) Analysis of body weight (BW), white adipose tissue (WAT) and liver weight (*n* = 31–37 mice per group for BW and 27–37 mice per group for the other samples). (**C**) Food consumption during the experimental protocol (*n* = 13–16 mice per group). (**D**) Energy efficiency of the body weight was calculated as mg gained divided by energy intake (Cal, *n* = 13–15 mice per group). (**E**) Respiratory exchange ratio obtained from the ratio of VCO_2_/VO_2_ (*n* = 2 mice per group). (**F**) Energy expenditure of individual mice (*n* = 2, 3 mice per group). Results were obtained from high-fat diet (HFD)-fed mice for 14 weeks and treated with Tb or placebo during the last 6 weeks. Data are presented as mean ± SD. **** *p* < 0.0001, *** *p* < 0.001, ** *p* < 0.01, * *p* < 0.05 HFD vs. HFD+Tb, Student’s *t*-test.

After eight weeks on the HFD, mice presented biochemical and metabolic alterations that characterize the state of obesity compared to mice given a control diet (CD, Figure S1A–E). After eight weeks of HFD feeding, obese mice were treated with tributyrin (Tb) or water for six weeks. We have previously demonstrated that Tb treatment has no effect on body weight or glucose metabolism in CD-fed mice [39]. In contrast, Tb-treated HFD-fed mice gained less body weight than mice treated with water after the end of the experimental protocol (Figure 1A,B). A reduction in subcutaneous WAT (Figure 1B) was observed in Tb-treated animals compared to the nontreated HFD group. These findings may be partly explained by the reduction in energy efficiency in the Tb-treated mice, even though there were no changes in food or caloric intake between the groups (Figure 1C,D). A decrease in the respiratory coefficient was also found in Tb-treated mice (Figure 1E). No significant difference in energy expenditure was observed between the experimental groups (Figure 1F).

**Figure 2:**
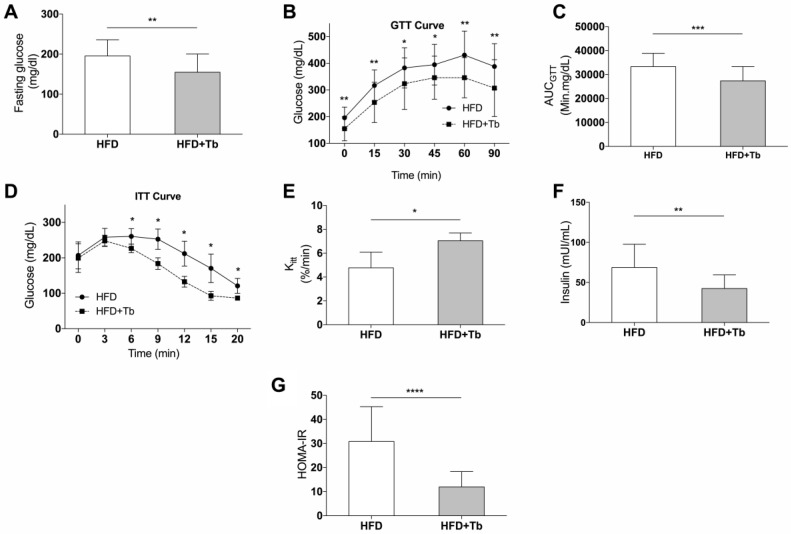
Tb administration improved glucose homeostasis in obese mice. (**A**) Glucose quantification in samples from 6 h fasted mice (*n* = 24–27 mice per group). (**B**) Glucose Tolerance Test was performed at the 12th week of the protocol (*n* = 23–27 mice per group). (**C**) Incremental area of blood glucose in the GTT (*n* = 23–27 mice per group). (**D**) Insulin Tolerance Test (ITT) was performed at the 13th week of the protocol (*n* = 4, 5 mice per group). (**E**) Rate of blood glucose disappearance (KITT) was calculated using data from ITT (*n* = 4 mice per group). (**F**) Serum insulin measured at the end of the protocol (*n* = 12–18 mice per group). (**G**) HOMA-IR was calculated from fasting insulin and fasting glucose (*n* = 12–18 mice per group). Data are presented as mean ± SD. **** *p* < 0.0001, *** *p* < 0.001, ** *p* < 0.01, * *p* < 0.05 HFD vs. HFD+Tb, Student’s *t*-test (A–F) or Mann–Whitney (D and G).

Treatment with Tb improved fasting glucose (Figure 2A), glucose tolerance (Figure 2B,C) and the response to insulin administration (Figure 2D,E). Additionally, a reduction in insulin concentration and improvement of insulin resistance, analyzed by HOMA-IR, were observed in Tb-treated mice (Figure 2F,G). Tb did not affect muscle metabolism, since soleus skeletal muscle metabolism after in situ stimulation with insulin was not different between the experimental groups (HFD vs. HFD+Tb, data not shown). This result is different from what we previously observed when we tested Tb for the prevention of obesity [39].

**Figure 3:**
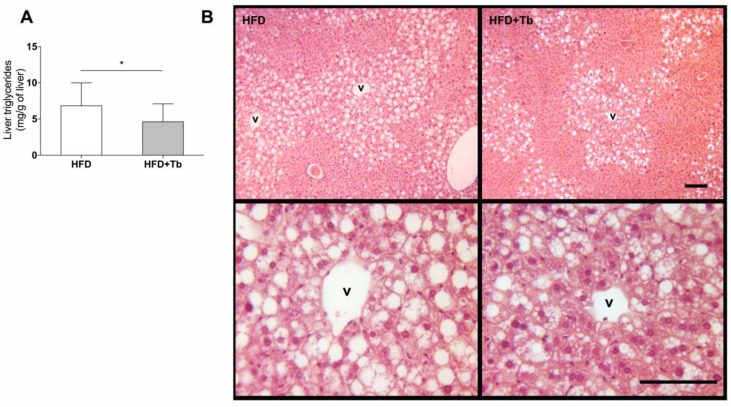
Tb treatment reduced the accumulation of triglycerides (TAG) in the liver and attenuated the signals of hepatic steatosis. (**A**) Triglycerides (TAG) were measured in liver samples from HFD-fed mice, and treated (HFD+Tb) or not (HFD) with Tb. Data are presented as mean ± SD (*n* = 12–17 mice per group). * *p* < 0.05 HFD vs. HFD+Tb, Student’s *t*-test. (**B**) Representative image of the liver histology (three mice from each group were analyzed). V, lobular central vein. Scale bars represent 100 μm.

Treatment with Tb significantly reduced serum concentrations of NEFA, triacylglycerol (TAG) and alanine aminotransferase (ALT) compared to the placebo group, but it had no effect on other lipid parameters, such as HDL, LDL, or total cholesterol (Table 1). We observed a reduction in liver weight (Figure 1B), ALT levels in the circulation (Table 1) and a decrease in TAG content and fat accumulation in the liver (Figure 3A,B). This indicated an improvement of hepatic function in Tb-treated mice compared to the control animals. 

**Figure 4:**
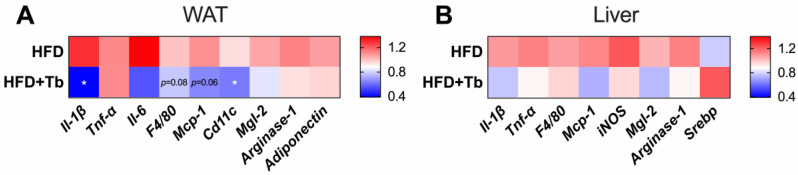
Tributyrin treatment reduced the expression of inflammatory markers. (**A**) mRNA expression of inflammatory markers, arginase-1 and adiponectin in the epididymal white adipose tissue (eWAT) (*n* = 6–9 mice/group). (**B**) mRNA expression of inflammatory markers, arginase-1 and Srebp in the liver (*n* = 5–9 mice per group). Data are presented as mean ± SD. * *p* < 0.05 HFD vs. HFD+Tb, Student’s *t*-test.

Next, we evaluated the expression of proinflammatory genes and macrophage markers in liver and WAT. In the liver, no significant difference was observed among the experimental groups, despite a general trend toward a reduction in inflammatory genes (Figure 4B). In contrast, the treatment of obese mice with Tb reduced the expression of inflammatory markers, including *Il-1β* and *Mcp-1* and of M1 macrophages (*Cd11c* and *F4/80*) in the WAT (Figure 4A). These results indicate that Tb may reduce inflammation in the WAT of obese animals. Adiponectin and sterol regulatory-element binding (Srebp) were also analyzed in WAT and liver samples, respectively, but no difference was observed.

### 3.2. Tributyrin Increases Serum Concentrations of Butyrate Independently of the Gut Microbiota

**Figure 5:**
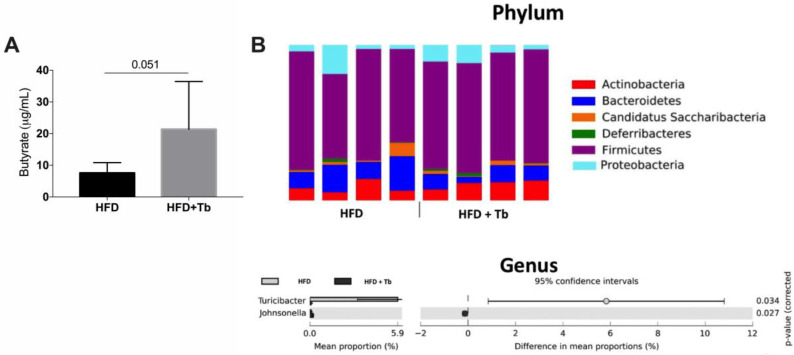
Tb treatment increased butyrate systemic concentrations with minimal effects on the microbiota. (**A**) Butyrate concentration was measured in blood samples at the end of the protocol. Data are presented as mean ± SD (*n* = 6, 7), Student’s *t*-test. (**B**) Analysis of fecal microbiota composition of HFD-fed mice treated or not with Tb showing phylum and genus (only genus statistically significant, *p* < 0.05; 95% of confidence intervals) distribution.

Tb administration increased the serum concentration of butyrate nearly three-fold compared to HFD-induced obese mice (21.4 ± 15.1 vs. 7.6 ± 3.3 μg/mL, Figure 5A). Next, we determined whether Tb could change the microbiota composition in HFD-fed mice. Using 16S metagenomic sequencing, we profiled the shifts in microbiome composition. At the phylum level, we observed that Tb treatment did not significantly alter community composition (Figure 5B). At the genus level, we only observed a significant difference between the experimental groups for two components of the microbiota (*Johnsonella* and *Turicibacter*, *p* < 0.05; 95% of confidence intervals, *n* = 4) of the 78 genera identified (Table S2). 

### 3.3. Tb-GPR109 Signaling Regulates Glucose Metabolism and Adipose Tissue Inflammation in Obese Mice 

**Figure 6:**
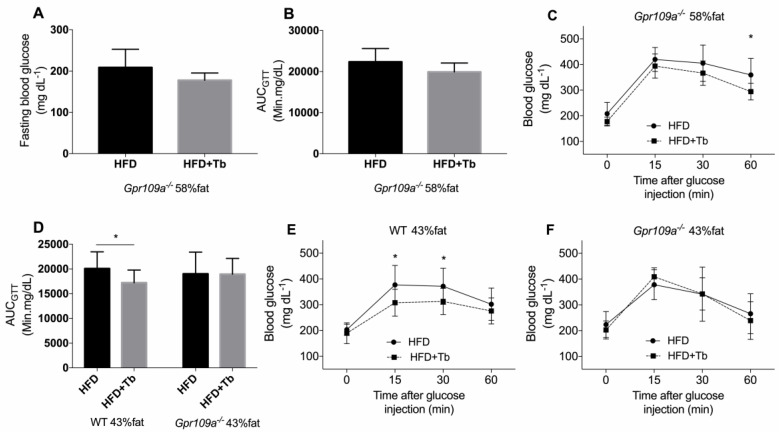
Tb improved glucose homeostasis in obese mice through a GPR109A-dependent mechanism. (**A**) Glucose quantification in samples from 6 h fasted *Gpr109a*^−/−^ mice (*n* = 8, 9 mice per group). (**B**) Incremental area of blood glucose (GTT) at the 12th week of the protocol in *Gpr109a*^−/−^ mice (*n* = 8, 9 mice per group). (**C**) GTT curves for *Gpr109a*^−/−^ mice are presented (*n* = 8,9 mice per group). (**D**) Incremental area of blood glucose (GTT) at the 12th week of the protocol in WT and *Gpr109a*^−/−^ mice (*n* = 9–14 mice per group). (**E**) GTT curves of WT mice are presented (*n* = 12–14 mice per group). (**F**) GTT curves of *Gpr109a*^−/−^ mice are presented (*n* = 9 mice per group). For this experiment, GTT was performed with the use of 1 g/kg glucose and a blood glucose meter (Accu-Check Performa^®^). Data are presented as mean ± SD (n is presented for each result). * p < 0.05 HFD vs. HFD+Tb. Student’s *t*-test.

Given Tb, as a prodrug of butyrate, prevents the development of metabolic and inflammatory alterations in HFD-fed mice [39]. Next, we wanted to determine whether the beneficial effects of Tb on glucose metabolism were mediated via “metabolite-sensing” G-protein coupled receptors. For that, we used mice deficient on GPR109A (*Gpr109a^−/−^* mice), a receptor that binds butyrate and mediates part of its effects on host tissues [11,12]. In contrast to the response observed in Tb-treated C57BL/6 mice, *Gpr109a^−/−^* mice did not show any significant change in glucose parameters after the administration of Tb (Figure 6A–C), indicating the participation of this receptor in the effect of Tb in obese mice. Then we asked whether the beneficial effects of Tb on glucose homeostasis were dependent on the intense model of diet-induced obesity used. We repeated the same analysis using HFD with less energy from fat (43% vs. 58%), which also is more relevant to humans [52]. In this experiment, we used *Gpr109a^−/−^* and *Gpr43^−/−^* mice +/− treatment with Tb. It is worth mentioning that GPR43 is a receptor activated by other SCFAs such as acetate and propionate [11,12]. Regardless of the HFD used, *Gpr109a^−/−^* mice did not show any significant change in glucose parameters after the administration of Tb (Figure 6D,F). In contrast, *Gpr43^−/−^* mice gained less body weight than placebo-treated mice, and showed a similar pattern of response to Tb treatment of the WT C57BL/6 mice (Figure S2). 

Regarding the biochemical parameters, we found that *Gpr109a^−/−^* HFD-fed mice (43% of energy from fat) presented an elevation of triglycerides (TAG) concentrations in serum compared with the WT mice (Table 2). This increase was attenuated by Tb treatment. We also observed a reduction in the NEFA concentrations caused by Tb treatment in WT and *Gpr109a*^−/−^ mice (Table 2). We measured the serum concentrations of hormones that are important for glucose homeostasis (glucagon-like peptide (GLP), gastric inhibitory peptide (GIP), leptin, insulin, peptide YY (PYY) and pancreatic polypeptide (PP)) in these mice. We found only a minor effect of Tb on PYY, which was independent of the genotype (Figure S3). 

**Figure 7:**
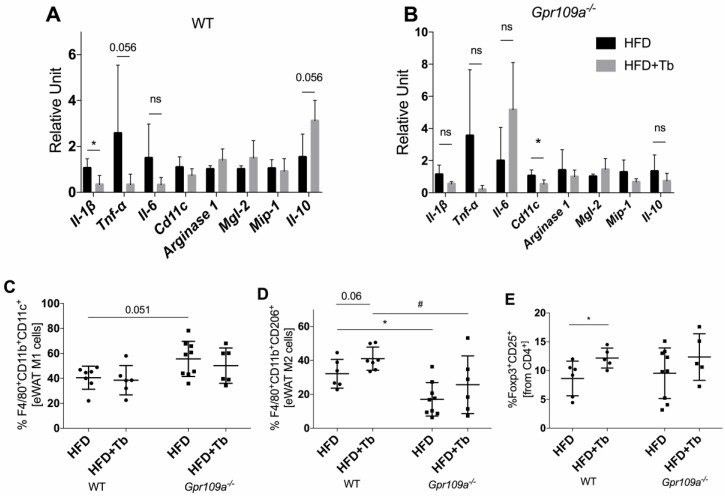
Tb attenuated the inflammation in WAT of obese WT mice through a GPR109A-dependent mechanism. (**A**,**B**) Effect of Tb treatment on the mRNA expression of inflammatory markers and cell markers (*n* = 4, 5 mice per group). * *p* < 0.05 HFD vs. HFD+Tb. Not significant (ns). (**C**) Frequency of F4/80^+^CD11b^+^CD11c^+^ M1 macrophages in WAT of obese mice treated or not with Tb (*n* = 6–9 mice per group). (**D**) Frequency of F4/80^+^CD11b^+^CD206^+^ M2 macrophages in WAT of obese mice treated or not with Tb (*n* = 6–9 mice per group). * *p* < 0.05 comparing HFD WT vs. HFD *Gpr109a*^−/−^ mice. # *p* < 0.05 comparing HFD+Tb WT vs. HFD+Tb *Gpr109a*^−/−^ mice. (**E**) Regulatory T cells (CD4^+^CD25^+^Foxp3^+^) were analyzed in WAT of WT and *Gpr109a*^−/−^ mice (*n* = 5–9 mice per group). * *p* < 0.05 comparing HFD vs. HFD+Tb. Results were analyzed using Mann–Whitney (A,B) or by two-way ANOVA and Bonferroni as post-test (**C**–**E**).

To elucidate the mechanisms behind GPR109A activation and the improvement in metabolic parameters, we analyzed the pattern of expression of inflammatory markers and infiltrating leukocytes in the WAT of WT and GPR109A knockout mice, treated or not with Tb. Similar to what we observed in Figure 4A, Tb treatment attenuated *Il1β,* an indication of reduced adipose tissue inflammation (Figure 7A). In contrast, *Gpr109a*^−/−^ mice treated with Tb showed no significant effect on inflammatory mediators such as *Tnf-α*, *Il1β*, *Il6* and *Il10* (Figure 7B). We observed a significant reduction in the expression of *Cd11c* in the WAT of *Gpr109a*^−/−^ obese mice. Interestingly, *Gpr109a*^−/−^ mice showed a significant increase in the frequency of M1 (F4/80^+^CD11b^+^CD11c^+^) macrophages (Figure 7C) and a significant decrease in the frequency of M2 macrophages (F4/80^+^CD11b^+^CD206^+^) in WAT (Figure 7D), thus indicating a shift toward a proinflammatory phenotype. Tb treatment did not affect the frequency of M1 macrophages in WT or *Gpr109a*^−/−^ mice (Figure 7C,D and Figure S4A). However, we observed an increase of M2 and regulatory T cells in this tissue after Tb treatment in WT (Figure 7D,E and Figure S4A,B). This latter effect was consistently observed in mice on both HFD diets (43% and 58%, not shown), but absent in *Gpr109a*^−/−^ HFD-fed mice (independently of the HFD used, Figure 7E). In addition, Tb effects on glucose tolerance (Figure 6D), NEFA concentrations (Table 2) and adipose tissue inflammation (Figure 7) observed in HFD-fed mice with 43% of energy from fat were present in the absence of a significant effect of Tb on body weight. This indicates that, at least for these parameters, Tb protective effects are independent of changes on body weight.

## 4. Discussion

In the present study, we demonstrated that Tb, a lipid that increases circulating levels of butyrate, attenuated body weight gain and the impairment of glucose metabolism of obese mice. Tb treatment was associated with the improvement of liver function and attenuation of eWAT inflammation. Mechanistically, we demonstrated that the effect on glucose metabolism and WAT inflammatory state was dependent on GPR109A activation.

Previous studies reported an increased Firmicutes/Bacteroidetes (F/B) ratio in gut microbial communities of genetically (ob/ob mice) or diet-induced obese mice [6,53]. This and other changes in microbiota composition and the consequent alteration in microbiota signaling to host cells are associated with the development of obesity and insulin resistance (reviewed by Khan et al., 2014) [54]. 

In our study, we observed that oral Tb was an effective way of increasing systemic concentrations of butyrate and improving glucose metabolism of obese mice. These effects were observed in the absence of major changes in microbiota composition, which is a key aspect to be considered for treatment of conditions associated with a dysbiotic microbiota, in which modifications of gut microbiota composition are not possible or which require longer periods of treatment, as appears to be the case for obese individuals [55,56]. 

A previous study has found that the addition of butyrate to an HFD (5% wt/wt) increased mice energy expenditure and oxygen consumption and decreased the RER in mice, suggesting amelioration in fatty acid oxidation [28]. These results were associated with an improvement of mitochondrial biogenesis and function in skeletal muscle and brown adipose tissue in butyrate-treated mice [28]. Consistent with this finding, we observed that Tb treatment decreased the energy efficiency and RER of obese mice, indicating that this treatment prevented further accumulation of lipids in tissues through an increase of energy expenditure and fatty acid oxidation, effects that mimic the changes observed with dietary butyrate supplementation.

Tb administration to obese mice reduced liver weight and the hepatic TAG content. These findings were associated with amelioration of systemic glucose homeostasis (lower fasting glucose, an improved glucose tolerance and higher insulin sensitivity). These protective effects on the liver of obese mice together with the capacity of Tb to attenuate liver steatosis and injury in other experimental models [57,58,59] indicate that this organ is a key target of Tb. Indeed, after Tb administration, high concentrations of butyrate have been observed in the portal vein and liver [58]. This SCFA can then act directly on hepatocytes, as demonstrated by a recent study [58], inhibiting histone deacetylases (HDACs) and, consequently, affecting the expression of important genes for hepatic function, such as carnitine palmitoyltransferase-1 (CPT-1A). The hepatic beneficial effects of tributyrin/butyrate can also be secondary to its action on other tissues such as the intestine [57] or WAT. 

A biochemical parameter that was consistently modified after the treatment of obese mice with Tb was the concentrations of circulating NEFA. A previous study suggested that NEFA concentrations in the circulation are more directly associated with liver injury than other parameters such as TAG deposition or TAG concentration in the circulation [60]. Other studies have also demonstrated a reduction in NEFA in mice treated with butyrate or Tb, associating this effect with inhibition of adipocyte lipolysis [39,61,62]. In our study, we found that, regardless of the genetics of mice (C57BL/6, *Gpr43*^−/−^ or *Gpr109a*^−/−^), Tb decreased NEFA in serum, supporting the premise that this effect was not related to the activation of GPR43 or GPR109A, as we expected. We hypothesize that the reduction of NEFA, and some of the effects on glucose metabolism are associated with the increased oxidation of fatty acids by the tissues induced by Tb, which is attributable to an improvement of mitochondrial function and efficiency, as previously demonstrated for butyrate supplementation [28,63]. 

GPR109A is expressed in adipocytes, immune cells [64] and intestinal epithelial cells and it is selectively activated by butyrate [65]. We observed that key metabolic and immune effects of Tb on obese mice were lost in the absence of this receptor, indicating that GPR109A mediates the Tb effects. In accordance with this, previous studies reported beneficial effects of other ligands of GPR109A on glycemic parameters. A clinical trial performed with GSK256073, a synthetic agonist of GPR109A, in T2DM patients found an improvement in glucose homeostasis and insulin sensitivity after treatment with this drug [66]. In addition, a recent study described that *Gpr109a*^−/−^ mice are more susceptible to the development of HFD-induced obesity compared to their controls and that administration of niacin (another ligand of GPR109a) attenuated the development of obesity in mice through a GPR109A-dependent mechanism [67]. 

Interestingly, we found that *Gpr109a*^−/−^ mice present more M1 macrophages and fewer M2 macrophages in the eWAT, indicating that their adipose tissue is in a more proinflammatory state compared to the WT mice. Tb treatment increased numbers of Tregs cells in WAT of WT mice, but not in *Gpr109a*^−/−^ mice suggesting that these cells may be relevant for the attenuation of inflammation observed after Tb treatment and also for the systemic beneficial effects of Tb (i.e., improvement of glucose metabolism). Previous studies have shown that mice with expanded Tregs numbers in the visceral WAT were protected against obesity-associated inflammation, insulin resistance and metabolic alterations, whereas the deletion of Tregs, especially from adipose tissue, abolished these effects [68,69]. Butyrate administration increased Tregs numbers in the colon of mice, an effect associated with HDACs inhibition [70,71]. In addition, Singh and collaborators showed that GPR109A signaling induces differentiation of cells into Treg and IL-10-producing T cells [65]. In this latter study, the authors focused on the cell populations present in the colon lamina propria and did not analyze the relevance of the receptor for Treg numbers in other tissues such as the adipose tissue. We found that butyrate increases Treg populations in the adipose tissue of obese mice via GPR109A signaling. This effect may contribute to the reduction of local inflammation and improvement of glucose homeostasis. Other mechanisms activated by Tb-GPR109A signaling may also contribute to the phenotype observed including the increased activity of brown adipose tissue and/or browning of WAT as previously reported [67]. 

## 5. Concluding Remarks and Perspectives

Tb is an alternative therapeutic approach to counteract the lack of microbial production of butyrate and restore immune and metabolic balance independently of the gut microbiota on obese individuals. The comprehension of Tb effects and mechanisms is a fundamental step toward the development of new therapeutic strategies for obesity treatment. It is worth mentioning that Tb is in use as a food additive and it has been shown to be well tolerated in humans [72]. These characteristics are important to evaluate the plausibility of proceeding to tests in other animal models and in the evaluation of safety in a potential clinical trial. However, this study has some limitations including the fact that we did not test lower doses of tributyrin. This is a critical point that will need to be addressed before tests in humans. 

## Figures and Tables

**Table 1:** Biochemical parameters measured in the serum from wild-type (WT) mice after 6 h of fasting treated or not with Tb.

	HFD	HFD+Tb
TAG (mg/dL)	64.4 ± 15.3 (*n* = 28)	54.7 ± 15.8 * (*n* = 30)
Cholesterol (mg/dL)	196.0 ± 33.9 (*n* = 25)	197.0 ± 36.3 (*n* = 26)
LDL (mg/dL)	48.4 ± 12.8 (*n* = 17)	48.1 ± 14.5 (*n* = 18)
HDL (mg/dL)	41.9 ± 8.5 (*n* = 16)	41.8 ± 6.2 (*n* = 16)
NEFA (mM)	1.160 ± 0.221 (*n* = 16)	0.900 ± 0.224 * (*n* = 17)
AST (U/mL)	17.19 ± 8.05 (*n* = 8)	16.53 ± 6.30 (*n* = 9)
ALT (U/mL)	11.77 ± 3.04 (*n* = 7)	7.85 ± 2.55 * (*n* = 9)

Data are presented as mean ± SD. * *p* < 0.05 HFD vs. HFD+Tb, Student’s *t*-test.

**Table 2:** Biochemical parameters measured in the serum from 6 h fasted WT and *Gpr109a*^−/−^ mice treated or not with Tb.

	WT		*Gpr109a* ^−/−^	
	HFD (*n* = 12, 13)	HFD+Tb (*n* = 13, 14)	HFD (*n* = 8, 9)	HFD+Tb (*n* = 9)
TAG (mg/dL)	47.8 ± 11.3	55.1 ± 15.4	101.4 ± 45.1	64.3 ± 23.7 *
Cholesterol (mg/dL)	125.3 ± 56.2	150.7 ± 59.8	177.5 ± 19.5	144.6 ± 42.8
LDL (mg/dL)	86.9 ± 43.0	105.5 ± 48.0	117.6 ± 12.9	100.2 ± 31.5
HDL (mg/dL)	28.1 ± 13.3	34.2 ± 13.9	36.1 ± 10.8	31.7 ± 14.5
NEFA (mM)	0.54 ± 0.12	0.40 ± 0.07 ***	0.56 ± 0.10	0.46 ± 0.10 *
AST (U/mL)	12.29 ± 2.13	12.71 ± 1.73	12.81 ± 2.10	11.83 ± 2.02
ALT (U/mL)	8.20 ± 1.48	8.21 ± 1.18	10.11 ± 1.94	10.04 ± 1.63

Data are presented as mean ± SD. *** *p* < 0.001, * *p* < 0.05 comparisons between HFD and HFD+Tb of the same genotype. Results analyzed using Student’s *t*-test.

## Supplementary Materials

The following are available online at https://www.mdpi.com/2073-4409/9/9/2007/s1, Figure S1: Mice on a HFD for 8-weeks presented a significant increase in body weight and an impairment in glucose metabolism compared to mice on control diet (CD, AIN93M), Figure S2: Tributyrin improved glucose homeostasis in obese mice through a GPR43 independent mechanism, Figure S3: Quantification of hormones in serum samples from WT, *Gpr43*^−/−^ and *Gpr109a*^−/−^ mice, Figure S4: Representative flow cytometry (FACS) plots of macrophages populations and Treg cells analysis performed with the WAT of WT and *Gpr109a*^−/−^ mice treated or not with Tb. Table S1: Sequence of the primers used in the study, Table S2: Proportion of genus (according to OTU-classification) levels in the microbiota of HFD-fed mice +/− Tb.
