# Supplementary material for: Tributyrin Attenuates Metabolic and Inflammatory Changes Associated with Obesity through a GPR109A-Dependent Mechanism
Source: Cells. 2020 Sep 1;9(9):2007. doi: 10.3390/cells9092007 (PMC7563536; doi:10.3390/cells9092007)
Supplement: Supplementary file 1 [file cells-09-02007-s001.pdf]

SUPPLEMENTARY FIGURES

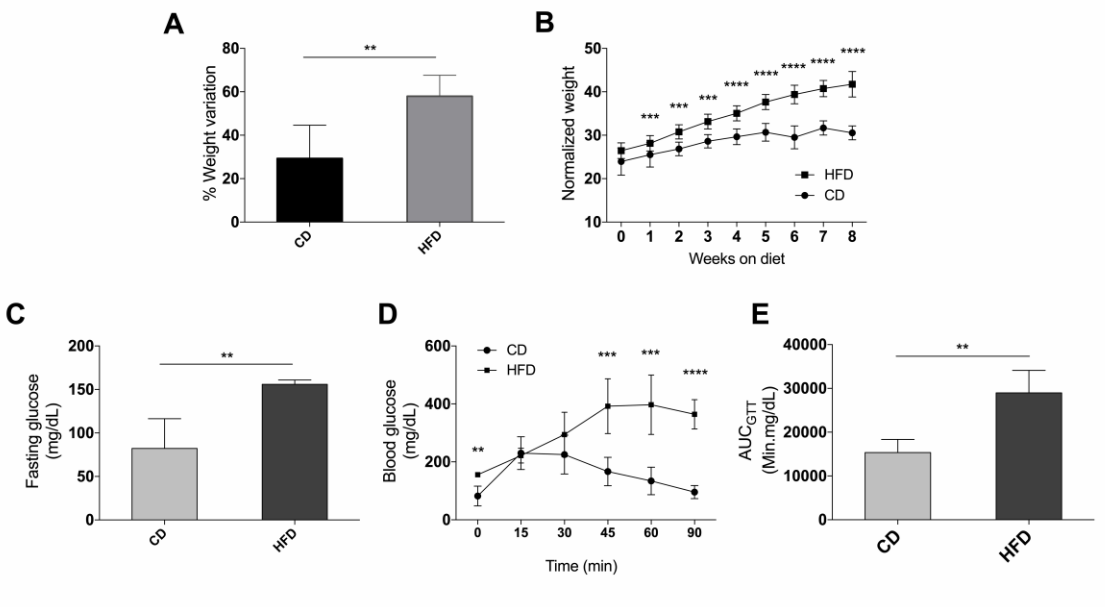

Figure S1. Mice on a HFD for 8-weeks presented a significant increase in body weight and an impairment in glucose metabolism compared to mice on control diet (CD, AIN93M). Body weight (BW) variation during the experimental protocol (A, B) (6-7 mice per group). Glucose quantification in samples from 6 h fasted mice (n = 4-6 mice per group) (C). Glucose Tolerance Test was performed at the 8th week of the protocol (n = 4-6 mice per group) (D). Incremental area of blood glucose in the GTT (E) (n = 5 mice per group). ****$p<0.0001$, ***$p<0.001$, **$p<0.01$ CD vs HFD, Student's t-test (B and D) or Mann-Whitney (A, C and E). Data are presented as mean ± SD.

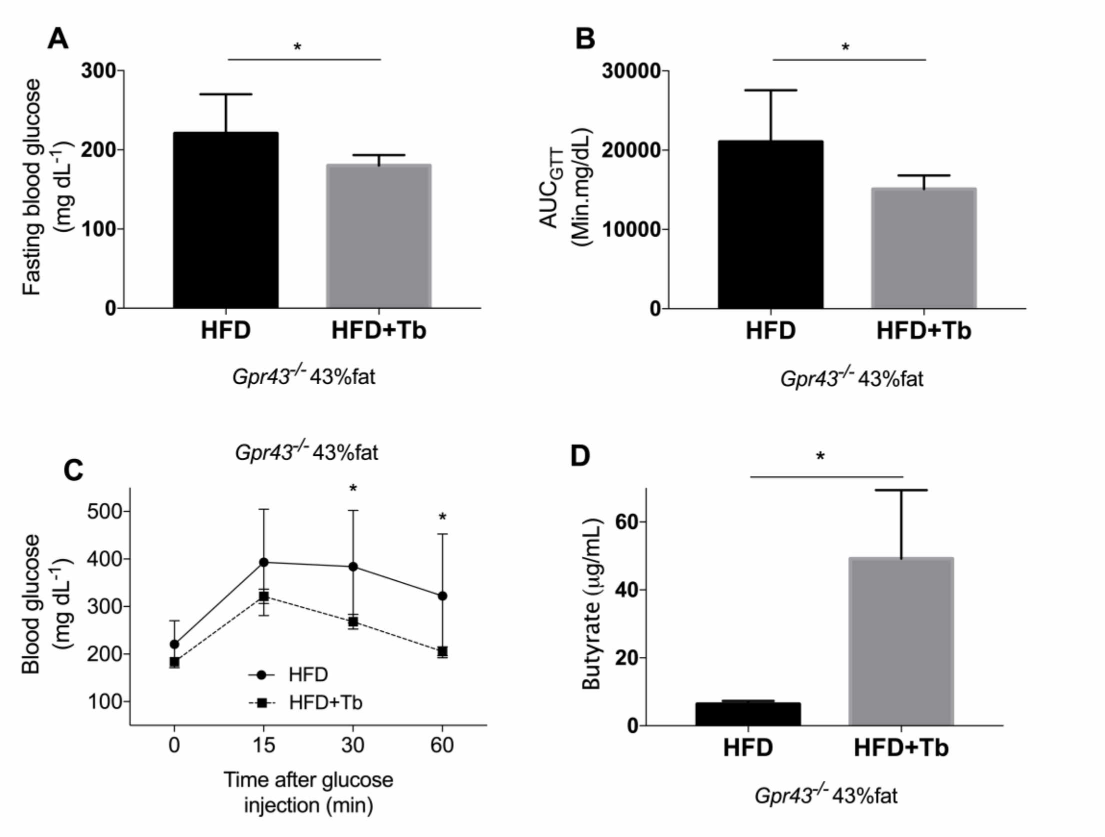

Figure S2. Tributyrin improved glucose homeostasis in obese mice through a GPR43 independent mechanism. (**A**) Glucose measurements in samples from WT and *Gpr43*$^{-/-}$ mice (n = 8-9 mice per group). (**B**) Incremental area of blood glucose (GTT) at the 12th week of the protocol in WT and *Gpr43*$^{-/-}$(n = 9 mice per group). (**C**) GTT curves are presented (n = 9 mice per group). (**D**) Butyrate concentrations in blood samples measured at the end of the protocol (n = 5 mice per group). *p <0.05. HFD vs HFD+Tb, Student's t-test (A, C and D) or Mann-Whitney (B). Data are presented as mean ± SD.

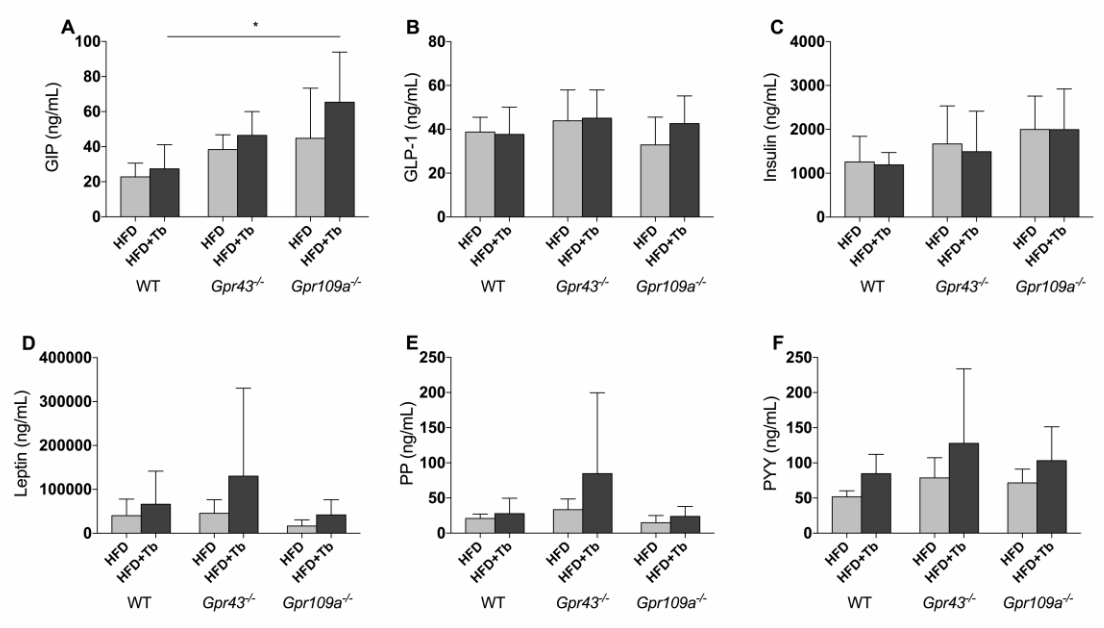

Figure S3. Quantification of hormones in serum samples from WT, *Gpr43*$^{-/-}$ and *Gpr109a*$^{-/-}$ mice. (**A**) Gastric inhibitory peptide (GIP), (**B**) Glucagon-like peptide-1 (GLP-1), (**C**) Insulin, (**D**) Leptin, (**E**) Pancreatic polypeptide (PP) and (**F**) Peptide YY (PYY) were measured in serum samples from WT, *Gpr43*$^{-/-}$ and *Gpr109a*$^{-/-}$ mice treated or not with Tb. Results are presented as mean ± SD (n = 5-10 samples per group). *$p<0.05$. Results analyzed by Two-way ANOVA, Bonferroni as post-test.

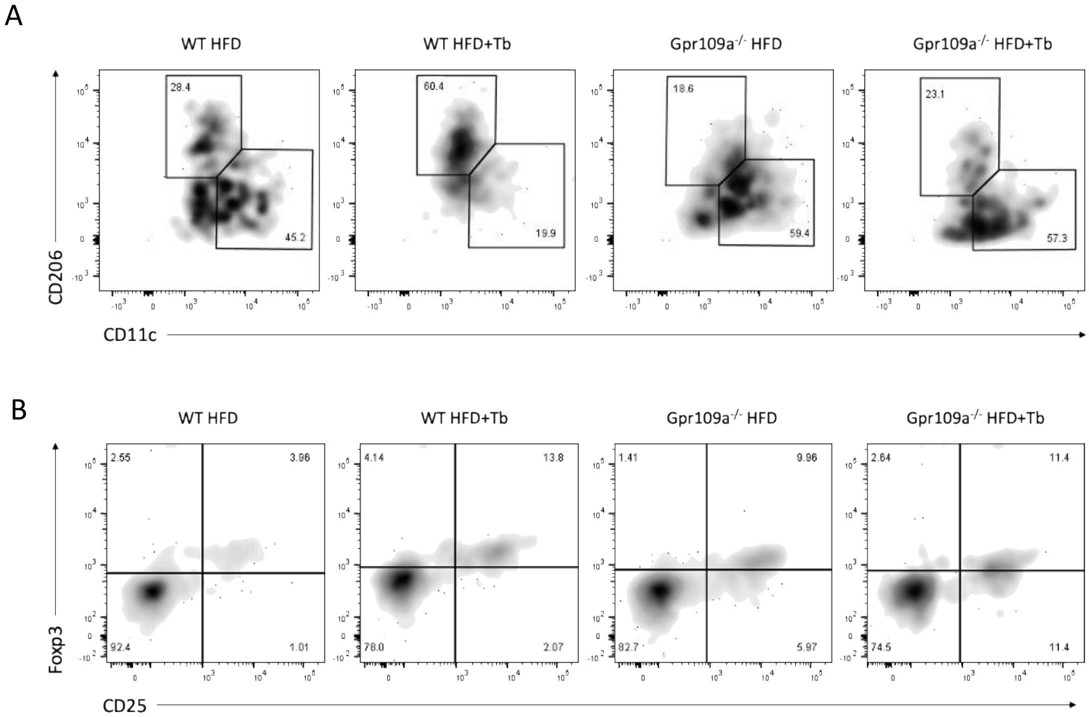

Figure 4. Representative flow cytometry (FACS) plots of macrophages populations and Treg cells analysis performed with the WAT of WT and *Gpr109a*$^{-/-}$ mice treated or not with Tb. (**A**) FACS plots for CD11c^{+} M1 and CD206^{+} M2 macrophages gated from F4/80^{hi}CD11b^{hi} population in the eWAT of mice treated or not with tributyrin. (**B**) CD25^{+}Foxp3^{+} regulatory T cells gated from CD4^{+} population in the eWAT of mice treated or not with tributyrin.

Table S1. Sequence of the primers used in the study.

Gene		Sequence
Adiponectin	sense	TCT TAA TCC TGC CCA GTC ATG C
	anti	TCC AAC ATC TCC TGT CTC ACC C
Arginase	sense	GGA AGA GTC AGT GTG GTG CTG G
	anti	CAG GAG AAA GGA CAC AGG TTG C
Cd11c	sense	AGC AGG TGG CAT TGT GGG AC
	anti	ACC TCT GTT CTC CTC CTC
F4/80	sense	CCT GAA CAT GCA ACC TGC CAC
	anti	GGG CAT GAG CAG CTG TAG GAT C
Il1β	sense	GGC AGC TAC CTG TGT CTT TCC C
	anti	ATA TGG GTC CGA CAG CAC GAG
Il6	sense	CGG AGA GGA GAC TTC ACA GAG
	anti	GGT AGC ATC CAT CAT TTC TTT G
iNOS	sense	CGG CAA ACC CAA GGT CTA CG
	anti	CAC CTG CTC CTC GCT CAA GTT C
Mcp-1	sense	ACC TGG ATC GGA ACC AAA TGA G
	anti	GAA GTG CTT GAG GTG GTT GTG G
Mgl-2	sense	TCA AGA ATT GGA GGC CAC TGC
	anti	AGT GGG CTG AGC TGG CTT TG
Srebp	sense	CAG TCA CCA GCT TCA GTC CAG G
	anti	CTG CTC AGG TCA TGT TGG AAA CC
Tnfa	sense	TCT TCT CAT TCC TGC TTG TGG C
	anti	CAC TTG GTG GTT TGC TAC GAC G
Ubiquitin c	sense	AGC CCA GTG TTA CCA CCA AG
	anti	ACC CAA GAA CAA GCA CAA GG
β2m	sense	CCC CAC TGA GAC TGA TAC ATA CG
	anti	CGA TCC CAG TAG ACG GTC TTG

Table S2. Proportion of genus (according to OTU-classification) levels in the microbiota of HFD-fed mice +/- Tb.

		HFD				HFD+Tb			
	TOTAL	SAMPLE 1	SAMPLE 2	SAMPLE 3	SAMPLE 4	SAMPLE 1	SAMPLE 2	SAMPLE 3	SAMPLE 4
Taxonomy									
Bacteria;Actinobacteria;Actinobacteria;Bifidobacteriales;Bifidobacteriaceae;Bifidobacterium	8.1%	6.1%	4.5%	12.7%	4.1%	6.2%	9.3%	10.4%	11.2%
Bacteria;Actinobacteria;Actinobacteria;Coriobacteriales;Coriobacteriaceae;Enterorhabdus	0.6%	0.7%	0.5%	0.7%	1.1%	0.4%	0.4%	0.6%	0.5%
Bacteria;Actinobacteria;Actinobacteria;Coriobacteriales;Coriobacteriaceae;Gordonibacter	0.0%	0.0%	0.0%	0.0%	0.0%	0.0%	0.0%	0.0%	0.0%
Bacteria;Actinobacteria;Actinobacteria;Coriobacteriales;Coriobacteriaceae;Olsenella	0.7%	0.9%	0.1%	0.4%	0.9%	0.3%	1.5%	0.6%	0.9%
Bacteria;Actinobacteria;Actinobacteria;Coriobacteriales;Coriobacteriaceae;Other	0.1%	0.1%	0.0%	0.1%	0.1%	0.1%	0.0%	0.1%	0.1%
Bacteria;Bacteroidetes;Bacteroidia;Bacteroidales;Bacteroidaceae;Bacteroides	0.9%	0.7%	2.1%	0.6%	1.7%	0.7%	0.2%	1.1%	0.4%
Bacteria;Bacteroidetes;Bacteroidia;Bacteroidales;Other;Other	2.7%	3.1%	3.1%	1.7%	6.4%	2.7%	0.9%	2.3%	1.6%
Bacteria;Bacteroidetes;Bacteroidia;Bacteroidales;Porphyromonadaceae;Barnesiella	3.9%	1.3%	6.8%	6.2%	5.0%	3.7%	1.2%	3.7%	3.6%
Bacteria;Bacteroidetes;Bacteroidia;Bacteroidales;Porphyromonadaceae;Odoribacter	2.4%	3.4%	3.9%	1.2%	2.8%	1.2%	1.2%	2.3%	2.9%
Bacteria;Bacteroidetes;Bacteroidia;Bacteroidales;Porphyromonadaceae;Parabacteroides	0.4%	0.2%	0.5%	0.3%	1.4%	0.4%	0.1%	0.6%	0.1%
Bacteria;Bacteroidetes;Bacteroidia;Bacteroidales;Porphyromonadaceae;Tannerella	0.1%	0.1%	0.1%	0.1%	0.1%	0.0%	0.0%	0.0%	0.1%
Bacteria;Bacteroidetes;Bacteroidia;Bacteroidales;Prevotellaceae;Alloprevotella	0.6%	0.1%	0.1%	0.2%	3.3%	0.3%	0.0%	0.2%	0.3%
Bacteria;Bacteroidetes;Bacteroidia;Bacteroidales;Prevotellaceae;Paraprevotella	0.0%	0.0%	0.0%	0.0%	0.0%	0.0%	0.0%	0.0%	0.0%
Bacteria;Bacteroidetes;Bacteroidia;Bacteroidales;Rikenellaceae;Alistipes	0.6%	0.5%	0.9%	0.6%	0.7%	0.4%	0.2%	0.6%	0.5%
Bacteria;Bacteroidetes;Bacteroidia;Bacteroidales;Rikenellaceae;Rikenella	0.4%	1.2%	0.3%	0.0%	0.7%	0.4%	0.1%	0.1%	0.2%
Bacteria;Candidatus Saccharibacteria;Saccharibacteria_genera_incertae_sedis;Saccharibacteria_genera_incertae_sedis;Saccharibacteria_genera_incertae_sedis;Saccharibacteria_genera_incertae_sedis	2.4%	1.2%	1.9%	0.7%	8.7%	2.3%	0.6%	2.7%	1.3%
Bacteria;Deferribacteres;Deferribacteres;Deferribacterales;Deferribacteraceae;Denitrovibrio	0.0%	0.0%	0.0%	0.0%	0.0%	0.0%	0.0%	0.0%	0.0%
Bacteria;Deferribacteres;Deferribacteres;Deferribacterales;Deferribacteraceae;Mucispirillum	0.7%	0.1%	2.1%	0.0%	0.3%	0.9%	1.8%	0.0%	0.2%
Bacteria;Firmicutes;Bacilli;Bacillales;Bacillales_Incertae Sedis XI;Gemella	0.1%	0.1%	0.2%	0.2%	0.4%	0.1%	0.0%	0.0%	0.0%
Bacteria;Firmicutes;Bacilli;Bacillales;Paenibacillaceae 1;Brevibacillus	0.0%	0.0%	0.0%	0.0%	0.0%	0.0%	0.0%	0.0%	0.0%
Bacteria;Firmicutes;Bacilli;Bacillales;Staphylococcaceae;Staphylococcus	0.0%	0.0%	0.0%	0.0%	0.1%	0.0%	0.0%	0.0%	0.0%
Bacteria;Firmicutes;Bacilli;Lactobacillales;Aerococcaceae;Globicatella	0.0%	0.0%	0.0%	0.0%	0.0%	0.0%	0.0%	0.0%	0.0%
Bacteria;Firmicutes;Bacilli;Lactobacillales;Enterococcaceae;Enterococcus	0.0%	0.0%	0.0%	0.0%	0.2%	0.0%	0.1%	0.0%	0.0%
Bacteria;Firmicutes;Bacilli;Lactobacillales;Lactobacillaceae;Lactobacillus	20.3%	30.0%	12.0%	30.2%	8.0%	13.6%	11.8%	25.8%	31.0%
Bacteria;Firmicutes;Bacilli;Lactobacillales;Lactobacillaceae;Other	0.0%	0.0%	0.0%	0.0%	0.0%	0.0%	0.0%	0.0%	0.0%
Bacteria;Firmicutes;Bacilli;Lactobacillales;Leuconostocaceae;Leuconostoc	0.3%	0.2%	0.2%	0.3%	0.6%	0.1%	0.2%	0.4%	0.2%
Bacteria;Firmicutes;Bacilli;Lactobacillales;Leuconostocaceae;Weissella	0.0%	0.0%	0.0%	0.0%	0.0%	0.0%	0.0%	0.0%	0.0%
Bacteria;Firmicutes;Bacilli;Lactobacillales;Streptococcaceae;Lactococcus	0.7%	0.6%	0.5%	0.7%	1.4%	0.5%	0.5%	0.9%	0.6%
Bacteria;Firmicutes;Bacilli;Lactobacillales;Streptococcaceae;Streptococcus	0.6%	0.3%	0.5%	0.5%	1.2%	0.4%	0.4%	0.7%	0.4%
Bacteria;Firmicutes;Clostridia;Clostridiales;Clostridiaceae 1;Clostridium sensu stricto	3.9%	4.8%	2.5%	5.7%	5.4%	2.2%	1.5%	6.6%	2.4%
Bacteria;Firmicutes;Clostridia;Clostridiales;Clostridiales_Incertae Sedis XIII;Anaerovorax	0.0%	0.1%	0.1%	0.0%	0.0%	0.1%	0.0%	0.0%	0.0%
Bacteria;Firmicutes;Clostridia;Clostridiales;Eubacteriaceae;Eubacterium	0.5%	0.2%	0.1%	0.2%	2.5%	0.4%	0.1%	0.5%	0.2%
Bacteria;Firmicutes;Clostridia;Clostridiales;Lachnospiraceae;Clostridium XlVa	2.0%	1.1%	2.7%	1.1%	1.6%	3.0%	2.3%	1.9%	2.2%
Bacteria;Firmicutes;Clostridia;Clostridiales;Lachnospiraceae;Clostridium XlVb	0.7%	0.9%	0.7%	0.5%	0.7%	0.9%	0.8%	0.6%	0.5%
Bacteria;Firmicutes;Clostridia;Clostridiales;Lachnospiraceae;Coprococcus	0.0%	0.0%	0.0%	0.0%	0.0%	0.0%	0.0%	0.0%	0.0%
Bacteria;Firmicutes;Clostridia;Clostridiales;Lachnospiraceae;Dorea	3.8%	3.1%	1.5%	3.0%	1.9%	9.9%	6.1%	1.5%	3.5%
Bacteria;Firmicutes;Clostridia;Clostridiales;Lachnospiraceae;Johnsonella	0.2%	0.1%	0.2%	0.1%	0.0%	0.3%	0.2%	0.2%	0.3%
Bacteria;Firmicutes;Clostridia;Clostridiales;Lachnospiraceae;Lachnospiracea_incertae_sedis	0.4%	0.3%	0.2%	0.6%	0.3%	1.1%	0.6%	0.3%	0.2%
Bacteria;Firmicutes;Clostridia;Clostridiales;Lachnospiraceae;Marvinbryantia	0.0%	0.0%	0.0%	0.0%	0.0%	0.1%	0.0%	0.0%	0.0%
Bacteria;Firmicutes;Clostridia;Clostridiales;Lachnospiraceae;Oribacterium	0.0%	0.0%	0.0%	0.0%	0.0%	0.0%	0.0%	0.0%	0.0%
Bacteria;Firmicutes;Clostridia;Clostridiales;Lachnospiraceae;Other	9.2%	6.8%	13.7%	6.3%	9.3%	13.2%	10.1%	7.6%	6.3%
Bacteria;Firmicutes;Clostridia;Clostridiales;Lachnospiraceae;Roseburia	0.0%	0.0%	0.1%	0.0%	0.1%	0.0%	0.1%	0.0%	0.0%
Bacteria;Firmicutes;Clostridia;Clostridiales;Lachnospiraceae;Ruminococcus2	0.0%	0.1%	0.0%	0.0%	0.0%	0.0%	0.0%	0.0%	0.0%
Bacteria;Firmicutes;Clostridia;Clostridiales;Other;Other	2.1%	2.1%	2.0%	2.5%	0.8%	5.0%	0.7%	1.8%	1.5%
Bacteria;Firmicutes;Clostridia;Clostridiales;Peptococcaceae 1;Peptococcus	0.1%	0.2%	0.2%	0.1%	0.2%	0.1%	0.1%	0.1%	0.1%
Bacteria;Firmicutes;Clostridia;Clostridiales;Peptostreptococcaceae;Clostridium XI	0.3%	0.8%	0.1%	0.3%	0.2%	0.2%	0.1%	0.7%	0.3%
Bacteria;Firmicutes;Clostridia;Clostridiales;Peptostreptococcaceae;Peptostreptococcus	0.0%	0.0%	0.0%	0.0%	0.0%	0.0%	0.0%	0.0%	0.0%
Bacteria;Firmicutes;Clostridia;Clostridiales;Ruminococcaceae;Acetanaerobacterium	0.0%	0.0%	0.0%	0.0%	0.1%	0.0%	0.0%	0.0%	0.0%
Bacteria;Firmicutes;Clostridia;Clostridiales;Ruminococcaceae;Anaerotruncus	0.6%	0.5%	0.4%	0.2%	0.4%	1.6%	0.9%	0.3%	0.4%
Bacteria;Firmicutes;Clostridia;Clostridiales;Ruminococcaceae;Butyricicoccus	0.0%	0.0%	0.1%	0.0%	0.0%	0.0%	0.0%	0.0%	0.0%
Bacteria;Firmicutes;Clostridia;Clostridiales;Ruminococcaceae;Clostridium III	0.0%	0.0%	0.0%	0.0%	0.0%	0.1%	0.0%	0.0%	0.0%
Bacteria;Firmicutes;Clostridia;Clostridiales;Ruminococcaceae;Clostridium IV	1.0%	0.8%	1.2%	0.8%	1.1%	1.3%	1.1%	0.7%	0.7%
Bacteria;Firmicutes;Clostridia;Clostridiales;Ruminococcaceae;Ethanoligenens	0.0%	0.0%	0.0%	0.0%	0.0%	0.0%	0.0%	0.0%	0.0%
Bacteria;Firmicutes;Clostridia;Clostridiales;Ruminococcaceae;Flavonifractor	1.5%	1.8%	1.4%	1.1%	1.0%	2.1%	2.5%	1.5%	0.8%
Bacteria;Firmicutes;Clostridia;Clostridiales;Ruminococcaceae;Hydrogenoanaerobacterium	0.2%	0.2%	0.3%	0.2%	0.1%	0.3%	0.4%	0.1%	0.1%
Bacteria;Firmicutes;Clostridia;Clostridiales;Ruminococcaceae;Oscillibacter	1.8%	1.9%	1.3%	1.4%	0.4%	2.5%	2.9%	2.4%	1.4%
Bacteria;Firmicutes;Clostridia;Clostridiales;Ruminococcaceae;Other	1.3%	1.3%	1.1%	1.3%	0.8%	1.7%	1.2%	1.5%	1.1%
Bacteria;Firmicutes;Clostridia;Clostridiales;Ruminococcaceae;Papillibacter	0.0%	0.0%	0.0%	0.0%	0.0%	0.0%	0.0%	0.0%	0.0%
Bacteria;Firmicutes;Clostridia;Clostridiales;Ruminococcaceae;Pseudoflavonifractor	1.3%	1.5%	1.2%	0.8%	0.3%	1.5%	3.1%	1.2%	0.8%
Bacteria;Firmicutes;Clostridia;Clostridiales;Ruminococcaceae;Ruminococcus	0.0%	0.0%	0.0%	0.0%	0.0%	0.0%	0.0%	0.0%	0.0%
Bacteria;Firmicutes;Erysipelotrichia;Erysipelotrichales;Erysipelotrichaceae;Allobaculum	12.1%	10.8%	5.6%	10.6%	10.5%	7.0%	22.7%	11.5%	17.9%
Bacteria;Firmicutes;Erysipelotrichia;Erysipelotrichales;Erysipelotrichaceae;Clostridium XVIII	0.0%	0.0%	0.0%	0.0%	0.0%	0.0%	0.1%	0.0%	0.0%
Bacteria;Firmicutes;Erysipelotrichia;Erysipelotrichales;Erysipelotrichaceae;Erysipelotrichaceae_incertae_sedis	0.0%	0.0%	0.0%	0.0%	0.0%	0.0%	0.0%	0.0%	0.0%
Bacteria;Firmicutes;Erysipelotrichia;Erysipelotrichales;Erysipelotrichaceae;Turicibacter	3.0%	5.7%	4.1%	3.5%	10.4%	0.1%	0.0%	0.2%	0.0%
Bacteria;Other;Other;Other;Other;Other	0.0%	0.0%	0.0%	0.0%	0.0%	0.0%	0.0%	0.0%	0.0%
Bacteria;Proteobacteria;Alphaproteobacteria;Rhizobiales;Bradyrhizobiaceae;Bradyrhizobium	0.0%	0.0%	0.0%	0.0%	0.0%	0.0%	0.0%	0.0%	0.0%
Bacteria;Proteobacteria;Alphaproteobacteria;Rhodospirillales;Rhodospirillaceae;Insolitispirillum	0.0%	0.0%	0.0%	0.0%	0.0%	0.0%	0.0%	0.0%	0.0%
Bacteria;Proteobacteria;Alphaproteobacteria;Rhodospirillales;Rhodospirillaceae;Other	0.0%	0.0%	0.0%	0.0%	0.0%	0.0%	0.0%	0.0%	0.0%
Bacteria;Proteobacteria;Betaproteobacteria;Burkholderiales;Sutterellaceae;Parasutterella	0.0%	0.0%	0.0%	0.0%	0.0%	0.0%	0.0%	0.0%	0.0%
Bacteria;Proteobacteria;Deltaproteobacteria;Desulfovibrionales;Desulfovibrionaceae;Bilophila	4.0%	2.3%	7.6%	1.8%	1.4%	6.9%	7.0%	3.4%	1.5%
Bacteria;Proteobacteria;Deltaproteobacteria;Desulfovibrionales;Desulfovibrionaceae;Desulfovibrio	0.2%	0.1%	0.4%	0.1%	0.1%	0.4%	0.4%	0.2%	0.1%
Bacteria;Proteobacteria;Deltaproteobacteria;Desulfovibrionales;Desulfovibrionaceae;Other	0.0%	0.0%	0.1%	0.0%	0.0%	0.1%	0.0%	0.0%	0.0%
Bacteria;Proteobacteria;Epsilonproteobacteria;Campylobacterales;Helicobacteraceae;Helicobacter	3.0%	1.7%	10.6%	0.6%	1.0%	3.3%	4.4%	1.0%	1.3%
Bacteria;Proteobacteria;Gammaproteobacteria;Enterobacteriales;Enterobacteriaceae;Escherichia/Shigella	0.0%	0.0%	0.0%	0.0%	0.1%	0.0%	0.0%	0.0%	0.0%
Bacteria;Proteobacteria;Gammaproteobacteria;Enterobacteriales;Enterobacteriaceae;Klebsiella	0.0%	0.0%	0.0%	0.0%	0.0%	0.0%	0.0%	0.0%	0.0%
Bacteria;Tenericutes;Mollicutes;Anaeroplasmatales;Anaeroplasmataceae;Anaeroplasma	0.0%	0.0%	0.0%	0.0%	0.0%	0.0%	0.0%	0.2%	0.1%
Bacteria;Tenericutes;Mollicutes;Mycoplasmatales;Mycoplasmataceae;Ureaplasma	0.0%	0.0%	0.0%	0.0%	0.0%	0.0%	0.0%	0.0%	0.0%
Bacteria;Verrucomicrobia;Verrucomicrobiae;Verrucomicrobiales;Verrucomicrobiaceae;Akkermansia	0.0%	0.0%	0.0%	0.0%	0.0%	0.0%	0.0%	0.0%	0.0%
